# Treating the Critically Ill with Radiotherapy: Lessons Learned from a Young Woman with Cardiac Angiosarcoma

**DOI:** 10.3389/fonc.2017.00029

**Published:** 2017-03-02

**Authors:** Emma C. Fields, Bryan Squires, Harry Lomas

**Affiliations:** ^1^Radiation Oncology, Virginia Commonwealth University, Richmond, VA, USA; ^2^Radiation Oncology, Wayne State University, Detroit, MI, USA

**Keywords:** cardiac angiosarcoma, radiotherapy, critically ill, chemotherapy, combined modality therapy

## Abstract

**Background:**

Treating patients who are critically ill with radiotherapy (RT) brings a unique set of challenges as it involves treating patients who require mechanical life support, unconventional positioning, and a multidisciplinary team approach to ensure safety. However, when the benefits of such treatment outweigh the risks, the challenges can be overcome, as demonstrated in this unique case of cardiac angiosarcoma.

**Case description:**

This is a case of a 42-year-old female with a right sided cardiac angiosarcoma who quickly developed cardiac tamponade and respiratory failure related to compression of her right heart by the tumor. She was treated with high dose single fraction RT initially and had a clinical response allowing further conformal RT with concurrent chemotherapy.

**Discussion:**

Managing critically ill patients requires creativity, improvisation, and careful consideration of existing evidence. Although limited, the data suggest that multi-modality therapy with a combination of surgery, RT, and chemotherapy provide the best outcomes.

## Summary

Treating patients who are critically ill with radiotherapy (RT) brings a unique set of challenges as it involves treating patients who require mechanical life support, unconventional positioning, and a multidisciplinary team approach to ensure safety. However, when the benefits of such treatment outweigh the risks, the challenges can be overcome, as demonstrated in this unique case of cardiac angiosarcoma.

Although rare, angiosarcoma is the most common primary cardiac malignancy and the majority occurs within the right atrial myocardium with invasion into the pericardium (1). Thus at presentation symptoms can include dyspnea, chest pain, and syncope related to pericarditis, pericardial effusion, and cardiac tamponade (2). Local control is important in this disease to improve these symptoms and prevent development of metastatic disease (3). However, in general, survival is poor with median survival between 12 and 36 months with multi-modality therapy (4, 5).

## Clinical Presentation

**Figure 1:**
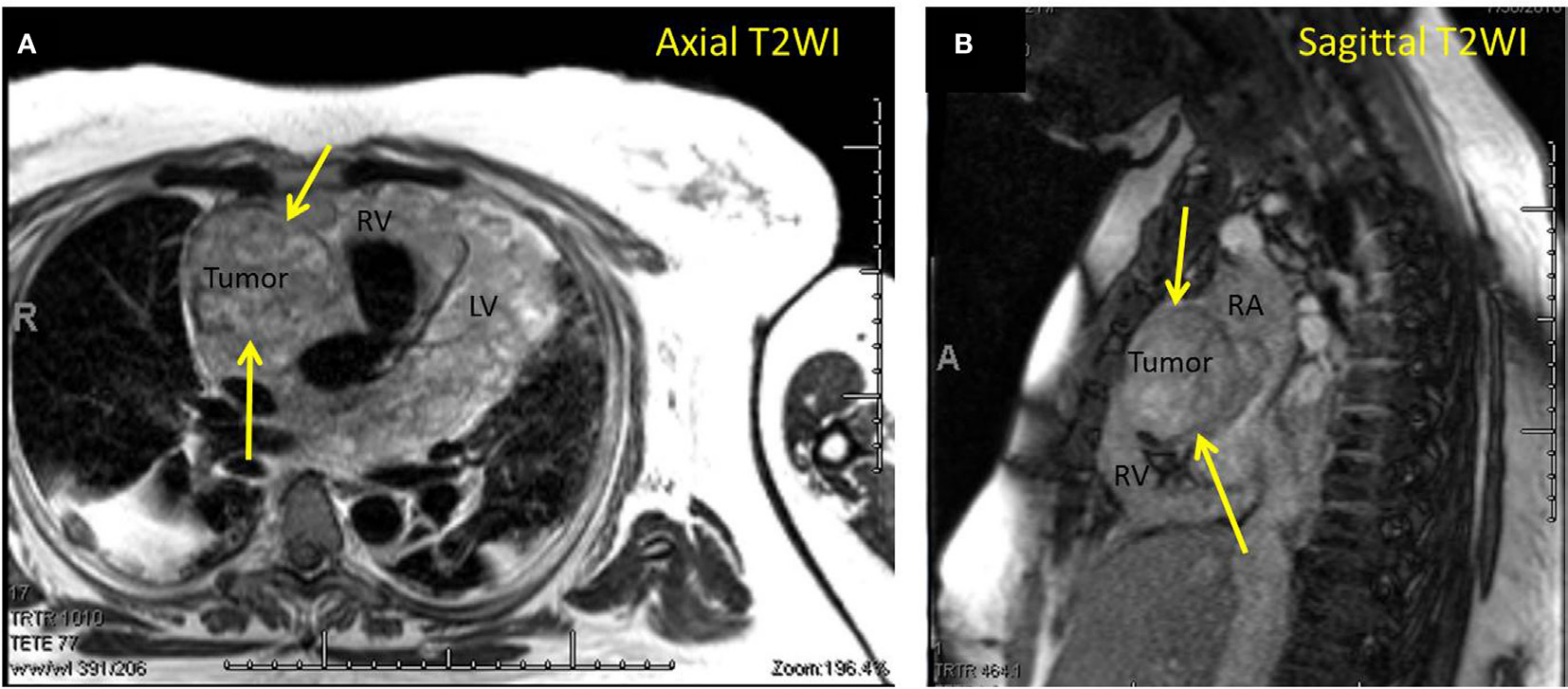
**(A)** Magnetic resonance imaging (MRI) axial T2-weighted image demonstrating cardiac angiosarcoma. Yellow arrows pointing to tumor. RV, right ventricle; LV, left ventricle. **(B)** MRI sagittal T2-weighted image demonstrating cardiac angiosarcoma. Yellow arrows pointing to tumor. RA, right atrium; RV, right ventricle.

A 42-year-old female presented with progressive dyspnea and bilateral lower extremity edema. Chest x-ray and a computed tomography scan of the chest revealed pericardial effusion. Trans-thoracic echocardiography demonstrated echogenic material within the effusion. During a pericardial window procedure, a pericardial mass adherent to the epicardium was discovered and biopsy confirmed cardiac angiosarcoma. Cardiac magnetic resonance imaging (MRI) showed a large heterogeneous mass in the right atrium and ventricle infiltrating the myocardium and pericardium (Figures 1A,B).

## Treatment

She was not a candidate for aggressive surgery due to her religious objection for receiving blood products. Therefore, it was decided to pursue concurrent chemoradiotherapy with external beam RT to a dose of at least 50 Gy in 25 fractions with weekly paclitaxel.

Several hours after initial supine simulation, she became dyspneic and orthopneic, necessitating intubation and ventilation. Due to tumor compression of the right ventricle, she could not lie flat without developing symptomatic bradycardia, and the decision was made to try a large single fraction emergently.

**Figure 2:**
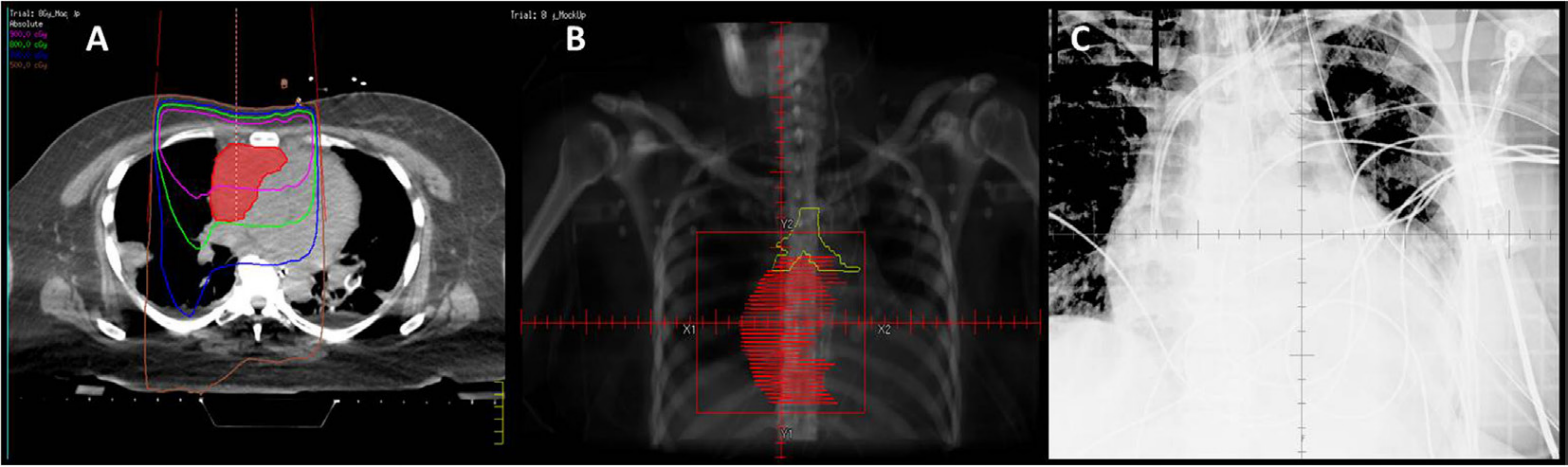
**(A)** Mock-up of clinical setup 8 Gy × 1 Gy, based on depth and field size calculations from prior CT simulation in supine position. **(B)** Mock-up of DRR for clinical setup with GTV (red). **(C)** kV image of AP setup.

Using the supine simulation CT scan, we constructed a single AP field based on tumor size and performed monitor unit calculations to a prescription point deep to the tumor for a dose of 8 Gy in a single fraction. We created a mock-up of this plan on the supine scan, but due to different patient positioning, this was in reality a clinical setup (Figure 2). Importantly, this could also have been done with a diagnostic CT or MRI if CT simulation had not yet been performed on the patient.

**Figure 3:**
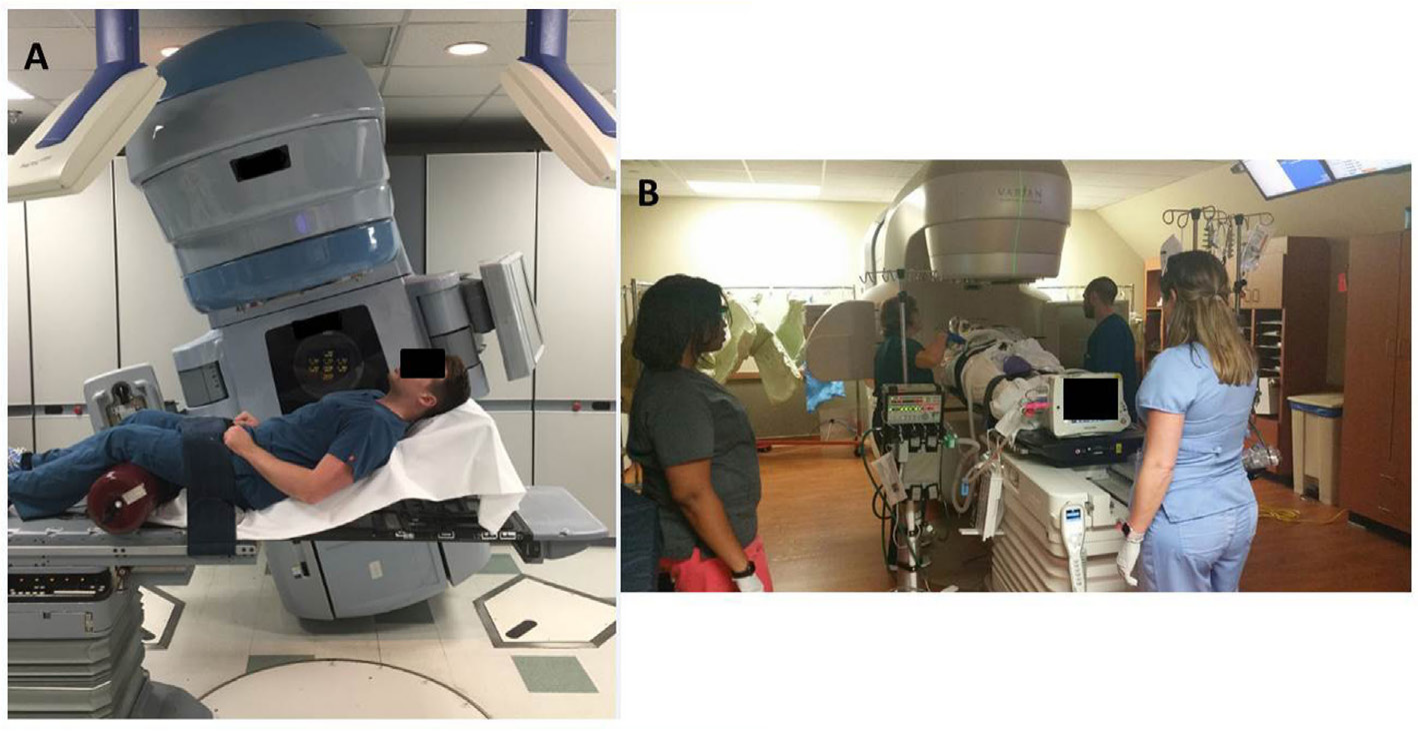
**(A)** Example of the patient positioning for 8 Gy fraction. The treatment couch and gantry are rotated to accommodate the patient’s inability to lie flat. **(B)** Example of extra staff and machines required in the room for daily treatments.

In order to at least obtain a port film in this position, with the couch at 90° and the gantry angled to provide an en-face photon AP field, the patient’s trunk needed to be flexed at 25° or less for the imager to clear without collision. The first attempt with this setup was unsuccessful because she was unable to lie at an angle below 45° without becoming tachypneic and hypoxic. The next day, the dose was successfully administered with this clinical setup (Figure 3A). This required the presence of the medical intensivist, cardiac ICU nurse, respiratory therapist, as well as the radiation oncologist, medical physicist, dosimetrist, and a multitude of therapists (Figure 3B).

**Figure 4:**
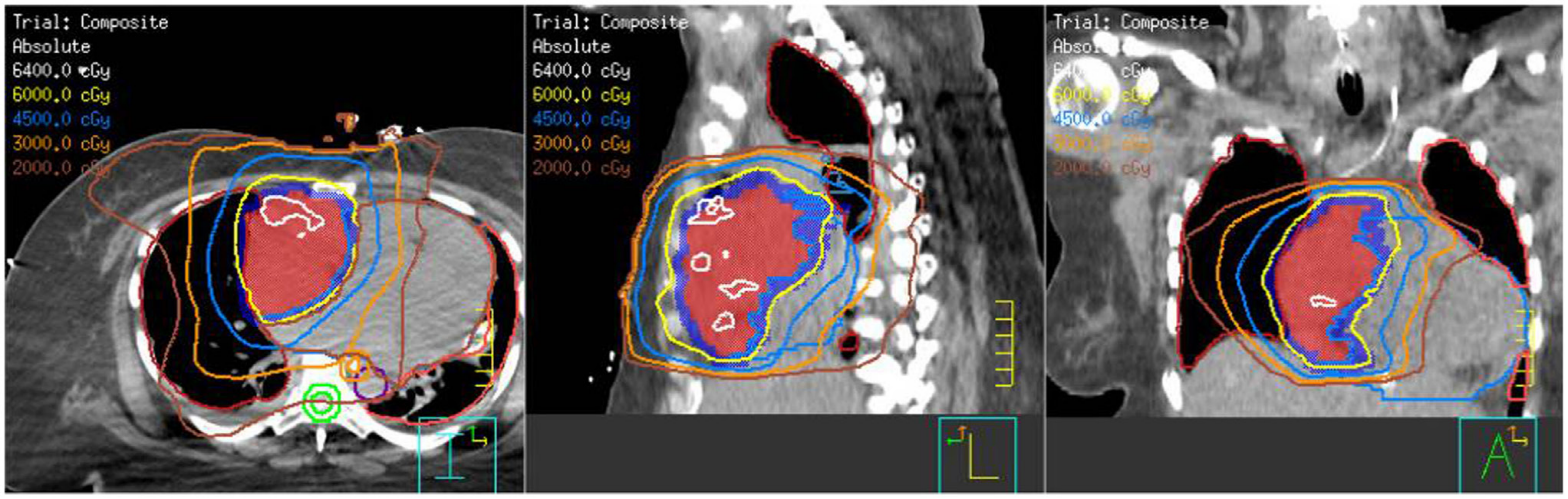
**Composite treatment plan showing the 8 Gy × 1 Gy with en-face photons plus the arc technique with the additional 50 Gy in 25 fractions**.

With the large fraction RT, her symptoms improved over a week, allowing re-simulation while lying at a 15° angle. An arc plan was created for an additional 50 Gy in 25 fractions (total dose of 58 Gy in 26 fractions) (Figure 4). She received her first treatment that day and subsequently initiated her first cycle of paclitaxel.

By the 10th day of fractionated treatment, the patient was able to be treated off of the ventilator with a tracheostomy collar. With the tumor burden decreasing, her right heart failure resolved and she had a cuff-less tracheostomy placed on the 17th day and was breathing room air. By the completion of her treatment, the patient was sitting up, talking, spending time with her daughters and awaiting discharge to a rehabilitation facility. She was seen at 1 month posttreatment and was without acute toxicity from the chemoradiation and with no clinical evidence of progression of disease.

## Discussion

Managing critically ill patients requires creativity, improvisation, and careful consideration of existing evidence. In this case report, we present the challenging situation of a young woman with cardiac angiosarcoma, a detailed assessment of our treatment techniques as well as a review of the published literature on this topic.

To date, there are several large retrospective studies documenting outcomes for patients with cardiac angiosarcomas (4, 5). However, it is difficult to evaluate outcomes based on these series as patients are included with varying stages of disease and non-uniform treatment algorithms. However, the primary conclusion is that combined modality therapy with the combination of surgery, chemotherapy, and RT, or the combination of chemotherapy and RT provide the best outcomes. Specifically, the addition of RT to either surgery or chemotherapy has been shown to improve progression-free survival (PFS) (4). The doses of RT used in these experiences are quite variable but have a median of 50 Gy (range 10–64 Gy) (Table 1). Based on the data, we chose to use 50 Gy in 25 fractions initially and with the addition of the 8 Gy in a single fraction ended up with 58 Gy in 26 fractions. For the additional 50 Gy, we used intensity modulated RT with arcs in order to respect the normal tissue tolerances for lung, esophagus, and spinal cord.

**Table 1:** **Radiotherapy (RT) doses and outcomes in cardiac angiosarcoma**.

Reference	Number of Pts	Chemotherapy	RT	Surgery	Outcome
Isambert et al. (4)	100 non-metastatic	90 patients, 90% with anthracycline-containing regimen	Range 10–64 Gy, median 50 Gy in a total of 24 patients	75 patients with complete resection in 13.3%	RT associated with improved progression-free survival
Randhawa et al. (5)	42	Most doxorubicin and ifosfamide total of 25 patients	15 patients, no doses	30 patients with complete resection in 6 patients	Multimodal therapy improved survival from 14.1 to 36.5 months
Aoka et al. (6)	1	Interleukin-2 adjuvant	64 Gy/16 fx with carbon-ion RT	None	1 year alive with disease
Takenaka et al. (7)	11 (sarcoma metastatic to heart from other sites)	3 patients (varied regimens)	25–60 Gy in 5–30 fx (in 7 patients)	None	Median OS with RT was 10.5 months compared to 3.5 without

Anthracyline-based chemotherapy has been the standard for soft-tissue sarcoma and is the primary chemotherapy used in the largest reported experience of patients with cardiac angiosarcoma (8–10). However, anthracyclines are known for their cardiac toxicity, which can be exacerbated with concurrent RT (11). More recently, taxanes have been used in patients with angiosarcoma due to the radiosensitivity, high clinical response rates, and favorable toxicity profile (12). The Phase II ANGIOTAX study using paclitaxel 80 mg/m^2^ on days 1, 8, and 15 every 4 weeks for two cycles in metastatic or unresectable angiosarcoma showed this regimen to be well-tolerated and provide 45% PFS at 4 months (12). This regimen has been used in primary cardiac angiosarcoma as well, with some long-term survivors (Table 2) (3, 13–16). This patient was treated with taxol 30 mg/m^2^ weekly during RT.

**Table 2:** **Taxol-based chemotherapy in cardiac angiosarcoma**.

Reference	Number of Pts	Chemotherapy	RT	Surgery	Outcome
Minichillo et al. (13)	1	Docetaxel 35 mg/m^2^ weekly	54 Gy/30 fx	None	2 years deceased of disease
Suderman et al. (15)	1	Docetaxel 25 mg/m^2^ weekly	Concurrent, no dose specified	None	16 months deceased with NED
Hata et al. (17)	1 (with bilateral lung nodules at presentation)	Paclitaxel 60 mg/m^2^ and carboplatin AUC 2 weekly	60 Gy/30 fx	Unresectable	5 months alive NED
Nakamura-Horigome et al. (3)	1	Docetaxel 25 mg/m^2^ weekly concurrent and adjuvant	42 Gy/21 fx	None	12 months alive NED
Jang et al. (16)	1	Docetaxel 25 mg/m^2^ weekly	50 Gy/30 fx	Initial resection with microscopic positive margins	32 months deceased of disease
Ram Prabu et al. (14)	1 (with bilateral lung nodules and pleural effusion at presentation)	Docetaxel 80 mg/m^2^ days 1, 8, 15 q 4 weeks	None	None	1 year alive NED

Despite the fact that rates of intensive care unit use is increasing in the United States and that malignancy is a common cause of airway obstruction, there is very little data on treating critically ill patients with RT (18–20). A retrospective study from London, ON, Canada, showed that of 26 patients who were intubated for malignant airway obstruction and treated with RT, 27% were successfully extubated and rates of extubation correlated with increased doses of RT. However, the study notes that these 26 patients are a small fraction of all patients intubated due to malignant obstruction and many may not even receive RT due to lack of referral, radiation oncologists’ reluctance to treat, or patient/family preference (21). The lack of referral and radiation oncologists’ reluctance to treat may be due to the scarcity of emergent indications for RT, as most patients are treated as outpatients. The case presented here demonstrates that with good team work and careful planning, these treatments can be accomplished successfully.

## Ethics Statement

This study was carried out in accordance with the recommendations of the VCU IRB with written informed consent from the patient and her family. All subjects gave written informed consent in accordance with the Declaration of Helsinki.

## Author Contributions

EF is the senior author on this paper. She treated this paper and helped with the construct and writing and editing. HL is a senior resident who helped with the images and formatting. BS is a fourth year medical student who did the research for the background section and first draft of writing.

## Conflict of Interest Statement

The authors declare that the research was conducted in the absence of any commercial or financial relationships that could be construed as a potential conflict of interest.
